# Heterogenic expression of stem cell markers in patient-derived glioblastoma spheroid cultures exposed to long-term hypoxia

**DOI:** 10.2217/cns-2017-0034

**Published:** 2018-04-30

**Authors:** Tine Rosenberg, Charlotte Aaberg-Jessen, Stine Asferg Petterson, Bjarne Winther Kristensen

**Affiliations:** 1 Department of Pathology, Odense University Hospital, Odense 5000, Denmark; 2 Department of ORL – Head & Neck Surgery, Odense University Hospital, Odense 5000, Denmark; 3 Department of Clinical Research, University of Southern Denmark, Odense 5000, Denmark; 4 Department of Nuclear Medicine, Odense University Hospital, Odense 5000, Denmark

**Keywords:** cancer stem cells, glioblastoma, glioblastoma subtypes, hypoxia, spheroid cultures, stem cell markers

## Abstract

**Aim:**

To investigate the time profile of hypoxia and stem cell markers in glioblastoma spheroids of known molecular subtype.

**Materials & methods:**

Patient-derived glioblastoma spheroids were cultured up to 7 days in either 2% or 21% oxygen. Levels of proliferation (Ki-67), hypoxia (HIF-1α, CA9 and VEGF) and stem cell markers (CD133, nestin and musashi-1) were investigated by immunohistochemistry.

**Results:**

Hypoxia markers as well as CD133 and partially nestin increased in long-term hypoxia. The proliferation rate and spheroid size were highest in normoxia.

**Conclusion:**

We found differences in hypoxia and stem cell marker profiles between the patient-derived glioblastoma cultures. This heterogeneity should be taken into consideration in development of future therapeutic strategies.

**Summary points**

- HIF-1α was rapidly induced in hypoxia, while it remained absent in normoxia until day 7, where a low expression appeared.
- VEGF showed a higher expression in hypoxia compared with normoxia.
- Low levels of CA9 were detected in all spheroid cultures.
- The expression of CD133 was in general increased in long-term hypoxia.
- No systematic hypoxia-induced up- or down-regulation of stem cell markers was identified in spheroid cultures being of different molecular subtype.
- The size of normoxic spheroids increased over 7 days – while the size of hypoxic spheroids was constant.
- The percentage of Ki-67-positive cells was reduced in long-term hypoxia.

Complicated therapeutic resistance mechanisms remain a crucial challenge in the development of new therapeutic strategies against glioblastomas, the most frequent and malignant primary brain tumor of which the median survival is only 14.6 months [1,2]. Presence of tumor hypoxia [3] and tumor stem cells [4–9] is related to this challenge together with the recent focus on the importance of pronounced tumor heterogeneity [10]. Glioma stem cells, which have also been described as tumor-initiating cells, are able to self-renew and thereby sustain glioblastoma growth. It has previously been demonstrated that both ionizing radiation therapy [4] and temozolomide chemotherapy enrich the glioma stem cell subpopulation [11]. This cell population is thus believed to be responsible for tumor maintenance and recurrence.

Research results from the last decade have suggested a firm link between hypoxia and tumor stem cells [3,12–14]. Most of these studies have focused on the effect of acute hypoxia, but recently the effect of long-term hypoxia has gained attention. Studies have revealed that long-term hypoxia drives nonstem cells toward a stem cell-like phenotype. Moreover, it may promote the self-renewal and maintenance of this undifferentiated phenotype [15–17]. Generally, previous studies have focused on either the acute or the long-term response while studies comparing these two responses are lacking. Moreover, though terms of ‘acute hypoxic response’ and ‘long-term hypoxic responses’ are frequently used, the hypoxic exposures leading to these responses *in vitro* remain controversial.

Glioblastomas are known for being highly heterogenic tumors, especially regarding gene expression and mutations. Based on this, four different glioblastoma subtypes (classic, mesenchymal, proneural and neural) have been identified and described [10]. Briefly, classical glioblastomas show a high expression of EGFR while mesenchymal glioblastomas show a high expression of mesenchymal and astrocytic markers (e.g., YKL40, CD44 and MERTK) and deletion of *NF1*. The expression of markers related to oligodendrocytic and proneural development (e.g., OLIG2, PDGFRA and TCF4) is increased in proneural glioblastomas. In addition, point mutations in the *IDH1* gene are often detected. Finally, neural glioblastomas show a higher expression of neuron markers (e.g., NEFL and GABRA1). Verhaak *et al*. suggested that these subtypes are important in terms of effect of therapy. To our knowledge, influence of hypoxia on the stem cell biology in different glioblastoma subtypes has only been sparsely described [18]. Therefore, the overall aim of this study was to set up a model focusing on the hypoxic time profile in glioblastomas of known molecular subtype. To do this, we cultured patient-derived spheroids characterized in our laboratory [19,20] in parallel under hypoxic and normoxic conditions. Spheroid cultures of mesenchymal, classical and proneural subtypes [21] were cultured in stem cell medium as 3D spheroids by which they are believed to maintain both the genotype and the phenotype of the primary tumor [22]. Cells were analyzed after 6, 12, 24, 48 and 72 h as well as after 7 days in either hypoxia or normoxia. By this, we aimed to include both the effects of acute and long-term hypoxia.

As markers of the hypoxic response, we used HIF-1α, CA9 and VEGF. HIF-1α is a key regulator in hypoxia [15,23]. The pH-regulating enzyme, CA9, was included since it often has been described to be induced by hypoxia [24–26]. Based on several studies showing that hypoxia promotes angiogenesis, VEGF was also included since HIFα is one of the important regulators of VEGF expression [15,23,27]. VEGF is potentially interesting due to the treatment strategies based on VEGF inhibitors. When inhibiting VEGF and thereby angiogenesis in the tumor it could potentially lead to more hypoxia, treatment resistance and more aggressive tumors due to a hypoxia-induced switch in cancer cell metabolism [28–31]. To monitor stem cell differentiation, we analyzed the expression of three stem cell markers, CD133 [32–38], nestin [33,37–43] and musashi-1 [35,38,42,44,45]. Expression of all markers was investigated by chromogenic immunohistochemical staining and quantified digitally in order to obtain precise less observer-dependent measurements [46,47].

## Materials & methods

### Spheroid cultures

In this study, the spheroid cultures T78 (mesenchymal subtype), T86 (classical subtype), T87 (proneural subtype) and T111 (mesenchymal subtype) were used. They are all glioblastoma short-term cultures with tumor stem cell properties established in serum-free medium in our laboratory as previously described [20,48,49]. These cultures showed repeated spheroid formation *in vitro* in serum-free medium, differentiation into cells expressing glial and neuronal markers in serum containing medium as well as formation of infiltrative tumors in immunodeficient mice upon orthotopic implantation. They were established from tissue obtained after informed patient consent at the Department of Neurosurgery at Odense University Hospital. In the present study, all spheroid cultures were used in passages 12–15.

### Cell culturing

Cells were cultured in a serum-free medium according to a previously described protocol [22,50]. Spheroids were cultured at 36°C humidified air containing 5% CO_2_ and 95% atmospheric air. When the average spheroid diameter reached 50 μm, half of the spheroids were transferred to a hypoxic incubator and cultured under hypoxic conditions (36°C humidified air containing 5% CO_2_, 2% O_2_ and 93% N_2_), whereas the other half were cultured under normoxic conditions (36°C humidified air containing 5% CO_2_ and 95% atmospheric air). Under these conditions, cells were harvested after 6, 12, 24, 48 and 72 h as well as 7 days. Spheroids cultured for 7 days were supplied with fresh conditioned medium at day 4. During the experiment, the oxygen level was estimated using an external calibrator on a daily basis and we did not observe any instability in the oxygen level in the incubator.

### Immunohistochemistry

Immunostaining was performed using a Dako Autostainer Universal Staining System (Dako A/S, Glostrup, Denmark). All reagents were obtained from Dako A/S, Denmark, and used as described by manufacturer's protocol. For more details, see previous publications [48,50,51]. Applied antibodies are listed in Table 1. Omission of primary antibodies served as negative controls as well as controls for nonspecific staining induced by the detection systems alone. Reactions were evaluated digitally by measuring the staining intensities of the spheroids using the VIS software (Visiopharm, Hørsholm, Denmark) [46,47,52]. Briefly, 20 randomly selected spheroids were marked as regions of interest. Dependent on the cellular location of the given protein, software classifiers for either membrane staining, nuclear staining or cytoplasmic staining were trained. Mean staining intensity and positive area fractions were calculated by the software within each regions of interest. Total immunopositivity was estimated as mean staining intensity × positive area fraction. The software classifiers were trained to identify specific staining reactions only. Potential background staining was below the cutoff level.

**Table 1.** **Antibodies used in this study.**

**Primary antibody**				**Detection system**	**Positive control**	**Ref.**

***Antibody***	***Supplier***	***Clone***	***Concentration***			
HIF-1α	BD Biosciences, NJ, USA	54	1 + 1000	PowerVision	Very large hypoxic spheroids of U87	–

Ki-67	Dako, Glostrup, Denmark	MIB-1	1 + 200	mEnVision	Human prostata	[53]

CA9	Novus Biological, CO, USA	Polyclonal NB100-417	1 + 1000	OptiView	Human stomach	[54]

VEGF	R&D Systems Inc., MN, USA	26503	1 + 1000	PowerVision	Human kidney	[55]

CD133	Miltenyi Biotec, Bergisch Gladbach, Germany	W6B3C1	1 + 40	CSAII	Human placenta	[56,57]

Nestin	R&D Systems Inc., MN, USA	196908	1 + 3000	mEnVision	Podocytes in human glomeruli	[58]

Musashi-1	MBL International, MA, USA	14H1	1 + 400	mEnVision	Human colon cells	[59]

### Statistics

For comparison of staining intensity one-way analysis of variance with Bonferroni correction was used. Unless otherwise stated data are shown as mean ± SEM, n = 20 and statistical significance */§ p < 0.05, **/§§ p < 0.01 and ***/§§§ p < 0.001. Statistical analyses were performed using Statistical Package for the Social Sciences software (SPSS, IBM Corporation, NY, USA).

## Results

### Spheroid area

**Figure 1.**
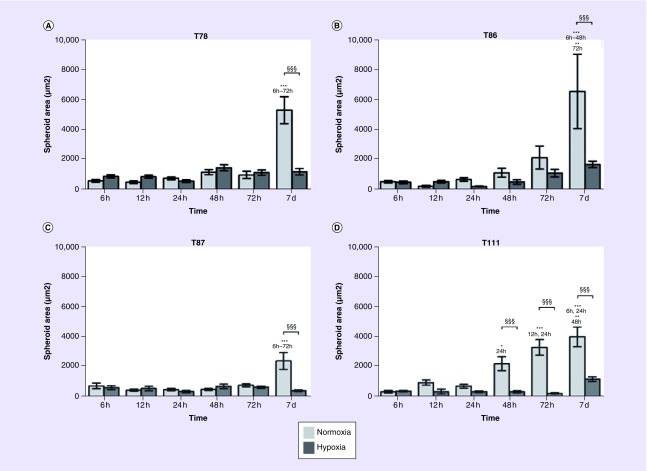
**Spheroid size over time.** Area of T78 **(A)**, T86 **(B)**, T87 **(C)** and T111 **(D)** spheroids after culturing in normoxia (light bars) or hypoxia (dark bars) in intervals between 6 h and 7 days. Statistical significances between normoxic and hypoxic samples are marked with §, while * indicates statistical significances within the same group (normoxia or hypoxia). T78: Mesenchymal subtype; T86: Classical subtype; T87: Proneural subtype; T111: Mesenchymal subtype.

No observable changes in spheroid area were seen in hypoxia during the 7 days. On the contrary, spheroid area was significantly increased after 7 days in normoxia; in T111, the significant increase was already seen after 48 h (Figure 1).

### Expression of Ki-67

**Figure 2.**
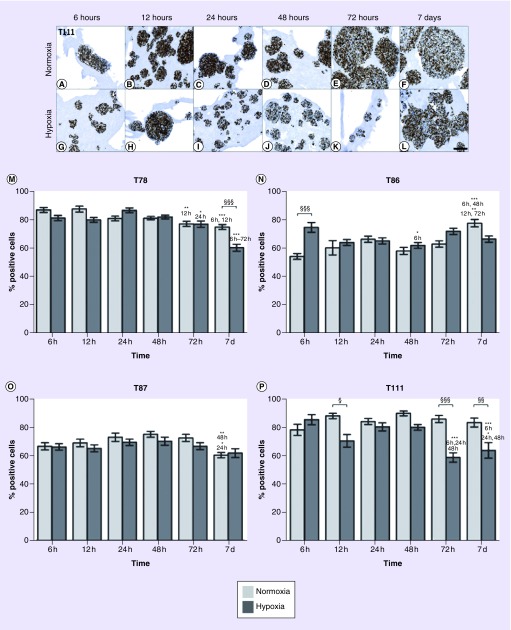
**Proliferation level over time.** The expression of Ki-67 showed a minor decrease in hypoxia compared with normoxia in T111 **(A–L).** Percentage of Ki-67 positive cells in T78 **(M)**, T86 **(N)**, T87 **(O)** and T111 **(P)** after culturing in normoxia (light bars) or hypoxia (dark bars) in time intervals between 6 h and 7 days. Statistical significances between normoxic and hypoxic samples are marked with §, while * indicates statistical significances within the same group (normoxia or hypoxia). Scale bar 100 μm. T78: Mesenchymal subtype; T86: Classical subtype; T87: Proneural subtype; T111: Mesenchymal subtype.

The Ki-67 level was estimated to be in the 60–90% range in the four spheroid cultures. A slight to moderate reduction of Ki-67-expressing cells was seen after 7 days in T78, T87 and T111. In T78 and T111 growing in hypoxia, the percentage of Ki-67-positive cells decreased from around 80–60% after 7 days (Figure 2M & P). In T78 and T111, the percentage of Ki-67-positive cells was lower in hypoxia compared with normoxia, especially after 7 days in hypoxia (p < 0.05 and 0.001; Figure 2A–M & P).

### Hypoxia-induced markers

**Figure 3.**
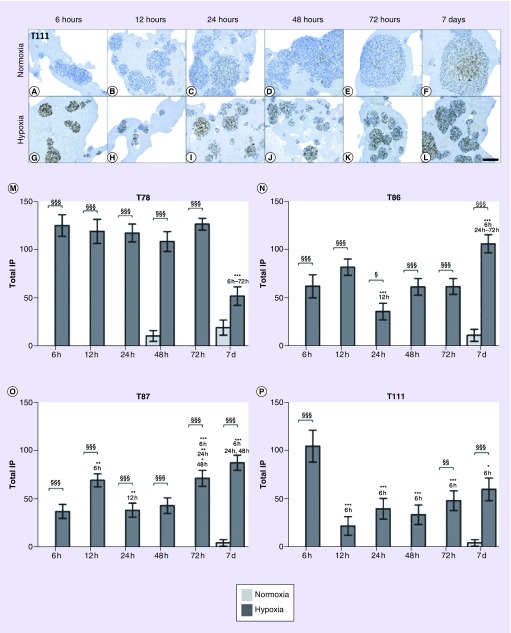
**HIF-1α level over time.** The upregulation is exemplified by T111 **(A–L).** The total IP of HIF-1α in T78 **(M)**, T86 **(N)**, T87 **(O)** and T111 **(P)** after culturing in normoxia (light bars) or hypoxia (dark bars) in time intervals between 6 h and 7 days. Statistical significances between normoxic and hypoxic samples are marked with §, while * indicates statistical significances within the same group (normoxia or hypoxia). Scale bar 100 μm. IP: Immunopositivity; T78: Mesenchymal subtype; T86: Classical subtype; T87: Proneural subtype; T111: Mesenchymal subtype.

HIF-1α showed a hypoxia-induced upregulation in all spheroid cultures being significant at almost all time points (Figure 3). The protein was rapidly induced in hypoxia, while it remained absent in normoxia until day 7, where a low expression was found.

**Figure 4.**
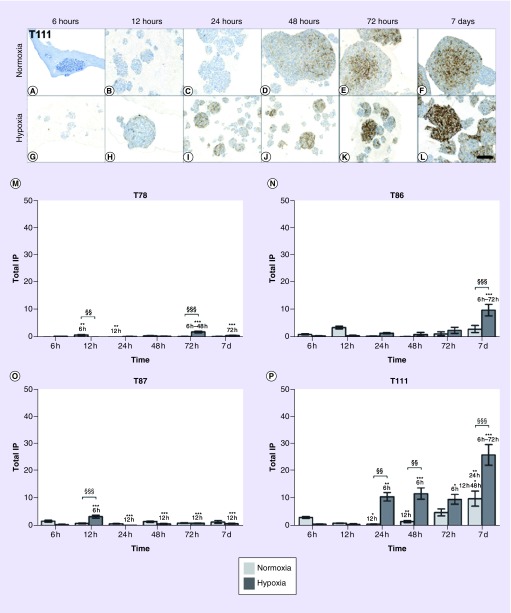
**CA9 level over time.** The expression of CA9 highly depended on the spheroid culture. In T86 **(N)** and T111 **(A–L & P)**, CA9 was induced after 6-h hypoxic exposure and increased until day 7. In T78 **(M)** and T87 **(O)**, the protein expression was hardly observable. Light bars normoxia, dark bars hypoxia. Total IP. Statistical significances between normoxic and hypoxic samples are marked with §, while * indicates statistical significances within the same group (normoxia or hypoxia). Scale bar 100 μm. IP: Immunopositivity; T78: Mesenchymal subtype; T86: Classical subtype; T87: Proneural subtype; T111: Mesenchymal subtype.

In general, only low levels of CA9 were observed in our spheroid cultures. However, higher levels were observed in hypoxia compared with normoxia. In T78 and T87, only a faint CA9 expression was observed (Figure 4M & O). In T86 and T111, CA9 was induced after 6 h of hypoxic exposure and from then on accumulating until day 7 (p < 0.001; Figure 4A–L, N & P). Toward the late time points, an increase in the protein expression was also seen in normoxia (Figure 4N & P).

**Figure 5.**
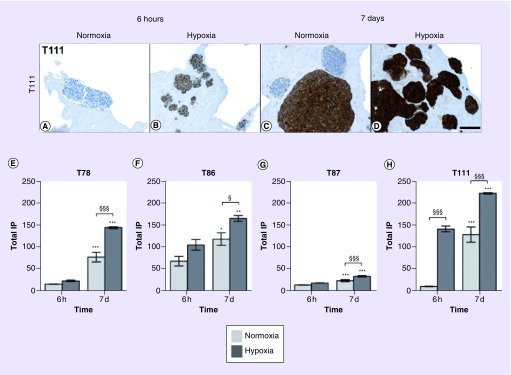
**VEGF level over time.** The expression of VEGF increased in response to hypoxia. In T111, VEGF was already significantly higher expressed in hypoxia after 6 h compared with normoxia **(A–D & H)**. In contrast, in T78 **(E)**, T86 **(F)** and T87 **(G)**, the protein expression was first significantly higher in hypoxia compared with normoxia after 7 days. Light bars normoxia, dark bars hypoxia. Mean intensity. Statistical significances between normoxic and hypoxic samples are marked with §, while * indicates statistical significances within the same group (normoxia or hypoxia). Scale bar 100 μm. T78: Mesenchymal subtype; T86: Classical subtype; T87: Proneural subtype; T111: Mesenchymal subtype.

The expression of VEGF was analyzed in spheroids cultured for either 6 h or 7 days in hypoxia and normoxia, respectively. In general, the protein expression was higher in hypoxia compared with normoxia for both time points. Moreover, a higher expression was generally seen after 7 days compared with 6 h for both normoxic and hypoxic spheroid cultures. Variations between the spheroid cultures were still seen. Compared with the other spheroid cultures, T86 showed a high protein expression after 6 h and the increase toward 7 days was relatively small (Figure 5F). Generally, VEGF was expressed at a low level in T87 (Figure 5G). In T111 (Figure 5A–D & H), the protein was rapidly induced by hypoxia but it still increased over time.

### Expression of stem cell markers

The expression of the three stem cell markers CD133, nestin and musashi-1 was investigated in spheroids cultured for 6 h and 7 days in hypoxia and normoxia, respectively. In general, the expression patterns varied widely between spheroid cultures, with no systematic hypoxia-induced upregulation of stem cell markers.

**Figure 6.**
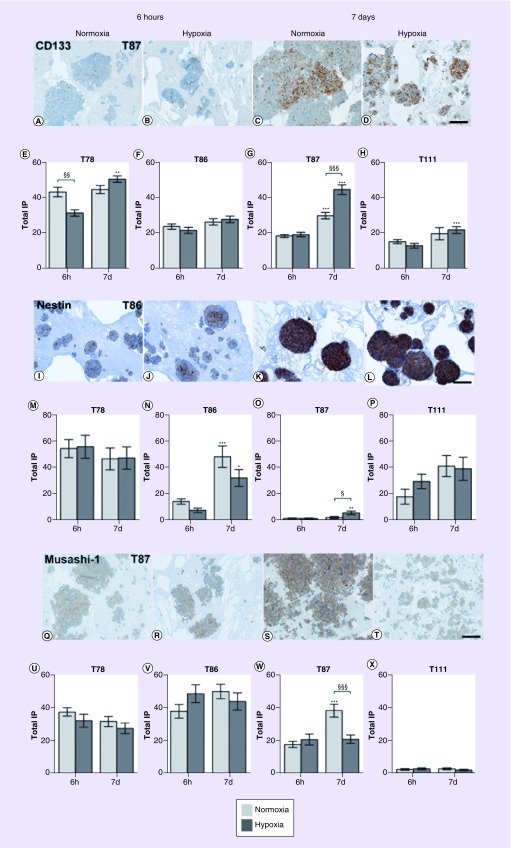
**Stem cell marker levels over time.** Hypoxia had no convincing effect on the expression of the stem cell markers CD133 **(A–H)**, nestin **(I–P)** and musashi-1 **(Q–X)**. Light bars normoxia, dark bars hypoxia. Total IP. Statistical significances between normoxic and hypoxic samples are marked with §, while * indicates statistical significances within the same group (normoxia or hypoxia). Scale bar 100 μm. IP: Immunopositivity.

The CD133 level increased from 6 h to 7 days in all spheroid cultures, but only significant in T78 (p < 0.01), T87 (p < 0.001) and T111 (p < 0.001) in hypoxia and for T87 (p < 0.001) in normoxia (Figure 6A–H).

The expression of nestin increased in the spheroid cultures T78, T87 and T111, although only significant in T86 (p < 0.05) and T87 (p < 0.001; Figure 6I–P).

There was no significant hypoxia-induced regulation of musashi-1 (Figure 6Q–X). The musashi-1 level increased from 6 h to 7 days in T87 in normoxia only.

## Discussion

In the present study, we aimed to elucidate differences in expression levels of hypoxia- and stem cell-related markers in glioblastoma spheroids of known molecular subtype. In summary, we found the expression of HIF-1α, CA9 and VEGF to increase in response to long-term hypoxia (7 days). Hypoxic spheroid cultures were generally smaller and had a lower proliferation rates compared with normoxic cultures. Finally, the stem cell markers CD133 and nestin were to some degree upregulated after 7 days in hypoxia.

Spheroids grown in normoxia for 7 days were significantly bigger than the corresponding spheroids grown in hypoxia. This was most pronounced for T78 and T111 and in line with the Ki-67 level being significantly reduced in hypoxia in these cultures at day 7. However, in general the proliferation only decreased modestly when the cells were exposed to long-term hypoxia. The prevention of a pronounced decrease in the proliferation could be explained by a general change in glucose metabolism in the tumor cells resulting in some proliferation, although the environment is hypoxic. Currently, it is well known that hypoxia plays multiple roles in cancer; however, the molecular changes at the cellular level are not fully elucidated. As a response to the hypoxic microenvironment, tumor cells have been shown to alter their glucose metabolism by diverting the glucose flux into the pentose phosphate pathway, where no oxygen is needed for the process (review in [60]). Because of this, cancer cells gain a proliferative advantage due to the limitations of oxidative damage by reactive oxygen species [28]. The cancer cells evade apoptosis, obtain genomic instability and unlimited proliferative potential [29].

HIF-1α was expressed in all spheroid cultures at all time points after exposure to hypoxia. In T86 and T87, there was a tendency for a late increase in the protein level. In T78 and T111, HIF-1α showed the highest expression after 6 h. The rapid induction of HIF-1α seen in this study supports previous results of HIF-1α to be induced after 2 [3,16] or 24 h of hypoxia [15,23]. The great variation in HIF-1α expression and different reaction patterns to hypoxia observed in our study could be explained by the pronounced tumor heterogeneity, which might be associated to different resistance mechanisms in tumors. Also the existence of four molecular subtypes described in the introduction might influence the varying HIF-1α expression. In addition, the spheroid cultures formed a variety of spheroids of different sizes varying between the spheroid cultures. Large spheroids will become more hypoxic explaining the variations observed among the cultures. For example, the T111 spheroids were small and the area remained constant after a few days of culturing. The decreasing HIF-1α expression observed in this spheroid culture after 6 h may partly be explained by the lack of increase in spheroid size. HIF-1α is upregulated as an acute response to hypoxia but since the spheroids stop increasing in size, a steady state in the microenvironment seems to be reached and the acute response decreases.

Relatively high differences in CA9 expression were seen between spheroid cultures, with the highest expression detected in T86 and T111. This confirms previous findings showing hypoxia-induced expression of CA9 after 6 h [61]. The protein CA9 is known to regulate the cellular pH and the expression was therefore expected to increase in all spheroid cultures. The upregulation of CA9 is commonly explained by upregulation of HIF-1α [24–26], but the CA9 expression and the HIF-1α expression do not necessarily follow each other. CA9 is upregulated as a response to the acidic environment due to the shift in glucose metabolism, but it has multiple functions. CA9 has been shown to be involved in cellular mechanisms such as proliferation, which may explain the variations observed between the spheroid cultures and why CA9 tends to increase under normoxic conditions for the T111 spheroid culture. Whether there is an association between the CA9 expression and the molecular subtype still needs to be elucidated in future studies. The late upregulation and the incipient expression in normoxic samples at the late time points (72 h and 7 days) are presumably due to an increasing spheroid size and thus a tendency of the central parts to become more hypoxic.

A distinct hypoxia-induced upregulation of VEGF was seen in all spheroid cultures, which are in line with other studies [15,23]. At both early and late time points, the expression was higher in hypoxia compared with normoxia. Moreover, the protein was upregulated over time in the individual spheroid cultures. The upregulation seen in normoxic cultures is most likely associated with spheroids being larger and partly hypoxic after 7 days. Though all spheroid cultures showed a hypoxia-induced upregulation, big variations between the cultures were seen. This might be explained by our spheroid cultures being of different molecular subtypes.

Hypoxia-induced upregulation of VEGF is generally thought to be induced by HIF-1α, which may also explain the increase in our setting. Although the expression of VEGF is mainly regulated by HIF-1α/STAT3 other regulators such as the protein tyrosine kinase Src has been identified [62].

Hypoxia has previously been demonstrated to promote the self-renewal of cancer stem cells and maintain the undifferentiated phenotype. Therefore, a clear tendency of hypoxia-induced upregulation of stem cell markers was expected, especially after 7 days [3,23,50,63]. All spheroid cultures expressed the stem cell markers CD133, nestin and musashi-1, but no systematic patterns were observed. For the stem cell marker CD133, the expression level increased significantly from 6 h to 7 days in T78, T87 and T111 in hypoxia. This is in line with previous studies demonstrating a hypoxia-induced increase in the expression of CD133 [3,18,50,64]. The expression of nestin also increased in hypoxia over time in T86 and T87. Though nestin has only been sparsely investigated in the context of hypoxia, these findings confirm previous studies carried out on both glioblastomas by our group [50] and on cochlear stem cells [65]. Regarding musashi-1, the highest expression was detected in T78, T86 and T87, whereas only a faint expression was observed in T111. No hypoxia-induced changes were observed in any spheroid cultures. To our knowledge, musashi-1 has not previously been investigated in the context of hypoxia, but recent studies suggest it as a possible prognostic marker in glioblastomas [45,52].

Although long-term increases in expression of stem cell markers were found, no markers showed the same initial expression level and increase over time in all four spheroid cultures. This may partly be explained by different subtypes: T78 and T111 are of mesenchymal subtype, T86 is of classical subtype and T87 is of proneural subtype [21]. Using The Cancer Genome Atlas database, Zarkoob *et al*. found a strong correlation between the mesenchymal and neural glioblastoma subtypes and *CD133* [66]. However, Brown *et al*. found the CD133 protein to be enriched in the proneural subtype [18]. In the present study, we found no significant variations between spheroid cultures of the three molecular subtypes, but inclusion of more stem cell markers will be needed to fully investigate this. SOX-2 would be a marker of high interest in such studies. In line with the hypoxia-induced upregulation of CD133 and nestin found in the present study, SOX-2 has previously been reported to be upregulated in response to hypoxia [67,68] and SOX-2 might therefore play an important role in hypoxia-induced biology of glioblastoma. Moreover, due to the pronounced tumor heterogeneity future studies should include more spheroid cultures representing each glioblastoma subtype in order to elucidate the subtype-dependent stem cell-related profile. Nevertheless, the potential subtype-dependent role of stem cell biology and association to the expression of stem cell markers may suggest that future investigations of stem cell-related mechanisms and hypoxia should be investigated to identify the responsible and targetable mechanisms.

Overall, the present study has shown that there are differences in hypoxic-, stem cell- and proliferation-marker profiles between the glioblastoma subtypes but also within each subtype making it an important but complex aspect to address in future treatment strategies. Moreover, it should be taken into consideration that it might be difficult to predict treatment response based on each subtype, since several studies have indicated that multiple molecular subtypes can be found in the same tumor underlining the importance of these hypoxic profiles [69,70].

## Conclusion

The present study has shown differences in hypoxia- and stem cell-marker profiles between different glioblastoma cultures. We have demonstrated heterogenous expression patterns in both short- and long-term perspectives. This should be taken into consideration in future glioblastoma research and in the development of new therapeutic strategies. Especially, regarding the anti-VEGF treatment leading to a more hypoxic microenvironment, this may be of critical importance.

## Future perspective

Treatment of glioblastomas remains a challenge. Targeting of, for example, VEGF as well as the stem cell aspects of the tumor biology has until now only been of limited success. We believe that a key to successful therapeutic strategies is a better knowledge and understanding of the heterogeneity of the tumor microenvironment. This may reveal different resistance mechanisms, which are associated with escape of some tumor cell populations, while other tumor cell populations are efficiently killed by the targeted therapy. Taking the tumor heterogeneity into account including critical microenvironmental features like hypoxia might therefore bring us closer to a better understanding of the complex biology of glioblastomas. Working in the era of precision medicine, this may suggest that novel therapeutics strategies should focus on microenvironmental aspects being critical in at least a part of glioblastomas. This may step-by-step increase the future survival for patients with glioblastomas.
